# Panoramic Infrazygomatic Crest Length: A Craniofacial Physiology-Based Analysis of Skeletal Class (I–III) and Sex in a Retrospective Cross-Sectional Study

**DOI:** 10.3390/diagnostics16101501

**Published:** 2026-05-15

**Authors:** Melis Büşra Aşkın, Ömer Can Manav, Ayşe Bulut

**Affiliations:** 1 Department of Orthodontics, Faculty of Dentistry, Yozgat Bozok University, 66100 Yozgat, Turkey; canmanav@hotmail.com; 2 Department of Physiology, Faculty of Dentistry, Yozgat Bozok University, 66100 Yozgat, Turkey; draysebulut@gmail.com

**Keywords:** infrazygomatic crest, panoramic radiography, panoramic length, skeletal Class I/II/III, temporary anchorage device, sexual dimorphism

## Abstract

**Objective**: This retrospective cross-sectional radiographic study evaluated panoramic infrazygomatic crest (IZC) length and compared values according to skeletal class and sex. The term panoramic IZC length was used to describe a two-dimensional linear measurement obtained on panoramic radiographs, rather than a three-dimensional bone corridor for miniscrew insertion. **Methods**: A total of 180 archived digital panoramic radiographs of patients aged 16–30 years were retrospectively collected and grouped equally by skeletal class and sex. Skeletal class was determined using previously recorded lateral cephalometric tracings based on ANB angle criteria: Class I, 0–4°; Class II, >4°; and Class III, <0°. Panoramic IZC length was measured bilaterally on calibrated panoramic images using ImageJ, and the bilateral mean was used for subgroup comparisons. Group differences were analyzed using one-way ANOVA, independent-samples *t*-tests, and two-way ANOVA. **Results**: Panoramic IZC length differed significantly across skeletal classes, with the highest values found in Class III and the lowest values found in Class II (*p* < 0.001). Mean panoramic IZC length was 5.81 ± 0.38 mm in Class III females and 6.16 ± 0.41 mm in Class III males, compared with 4.99 ± 0.36 mm in Class II females and 5.51 ± 0.40 mm in Class II males. Males showed significantly greater values than females (*p* < 0.01). The sex-by-class interaction was significant (*p* = 0.04). Intra-observer repeatability was excellent (ICC = 0.95). **Conclusions**: Panoramic IZC length varies according to skeletal class and sex, with higher values in Class III individuals and males. These findings provide preliminary comparative morphometric information from routinely available panoramic radiographs. However, panoramic IZC length should not be interpreted as a three-dimensional insertion corridor, and CBCT remains necessary when comprehensive anatomical planning, sinus proximity assessment, or extra-alveolar miniscrew trajectory evaluation is required.

## 1. Introduction

The infrazygomatic crest (IZC) is an extra-alveolar maxillary region located along the zygomatic buttress, typically described between the zygomatic process of the maxilla and the posterior alveolar ridge and in close anatomic relation to the maxillary sinus. Because IZC anchorage is positioned outside the dental root corridor, temporary skeletal anchorage devices (TSADs) placed in this area can facilitate large-magnitude tooth movements with minimal dependence on patient compliance, including total arch distalization, anterior en masse retraction, and vertical control mechanics [1,2,3].

The anatomical suitability of the IZC for TSAD placement is not uniform across individuals. CBCT-based studies have shown clinically relevant inter-individual variability in IZC bone depth and thickness, and this variability has been associated with craniofacial morphology, vertical facial growth pattern, skeletal classification, age, and sex [4,5,6,7]. In particular, investigations mapping IZC dimensions across different craniofacial patterns highlight that insertion “windows” and available bone may shift depending on facial type and skeletal relationships, reinforcing the need for individualized assessment rather than assuming a single universally optimal site [4,5,6,7].

Clinical relevance of skeletal pattern and sex in IZC assessment arises from their potential to shift the available bone envelope and the location of more favorable insertion “windows” for extra-alveolar anchorage. CBCT-based mapping studies have reported that IZC bone availability varies across craniofacial morphologies and facial types, and that trajectory-related parameters (insertion height and angulation) materially influence the thickness/depth profile encountered along the planned path [4,5,6,7,8,9,10,11,12]. Because IZC screws are frequently used to deliver distalization, en masse retraction, and vertical control mechanics, subgroup-specific anatomic tendencies (rather than deterministic rules) can be useful for preliminary appraisal and for deciding when a three-dimensional assessment is clinically warranted [1,2,3,13].

Imaging-based evidence has also emphasized that safe and predictable placement depends on local morphologic conditions (depth/thickness) and proximity to adjacent anatomical structures, most notably the maxillary sinus, in addition to insertion angulation and vertical height selection [5,6,7,8]. Accordingly, guideline-oriented CBCT studies have proposed practical insertion parameters (e.g., height from the occlusal plane and angulation ranges) to maximize cortical engagement while reducing the risk of iatrogenic complications [8,9]. More broadly, the orthodontic miniscrew literature underscores that anatomic constraints and regional risks should be explicitly considered during site selection and planning [14].

Although CBCT provides three-dimensional visualization for detailed anatomic planning, it is not always feasible as a routine imaging modality in all settings. Panoramic radiography remains widely available in orthodontic records and is frequently used for screening and retrospective assessments, but its linear measurements are affected by magnification, distortion, and head-positioning sensitivity [15,16,17]. Where panoramic archives are used for quantitative comparisons, measurement approaches should be interpreted with these modality-specific limitations in mind and supported by calibration and standardized landmark definition when possible [15,16,17].

From a radiation-protection perspective, panoramic radiographs may still have practical value when they are already available as part of routine orthodontic records. In such cases, standardized panoramic measurements can offer preliminary comparative information without requiring additional radiation exposure. This potential value should be interpreted within the known limitations of panoramic imaging and should not be considered a substitute for CBCT-based planning when detailed three-dimensional assessment is clinically required.

Previous studies have shown that panoramic radiographs can provide reproducible linear measurements under standardized positioning, calibration, and landmark definition conditions [15,16,17]. However, reproducibility should not be equated with three-dimensional anatomical validity. Panoramic measurements remain vulnerable to projection-related distortion, magnification, and superimposition, especially in posterior maxillary regions. Therefore, in the present study, panoramic radiography was used for retrospective two-dimensional morphometric comparison rather than for direct evaluation of the true IZC bone envelope or miniscrew insertion path.

Therefore, the present study aimed to evaluate panoramic IZC length on calibrated digital panoramic radiographs and to analyze differences according to skeletal classification and sex. In this study, panoramic IZC length refers to a standardized two-dimensional radiographic distance measured on panoramic images, not to the three-dimensional bone envelope or oblique extra-alveolar insertion corridor used for IZC miniscrews. By using existing panoramic records, this study sought to provide preliminary morphometric reference information while maintaining the distinction between panoramic radiographic appearance and CBCT-based anatomical planning.

## 2. Materials and Methods

Ethical approval for this retrospective study was obtained from the Institutional Ethics Committee (Approval No: 2026-GOKAEK-263_2026.02.04_23; Date: 4 February 2026). All radiographs were anonymized prior to analysis, and informed consent was waived in accordance with institutional regulations.

A total of 180 digital panoramic radiographs were retrospectively collected from patients aged 16 to 30 years, selected from clinical records between January 2020 and December 2024.

### 2.1. Participant Flow

**Figure 1:**
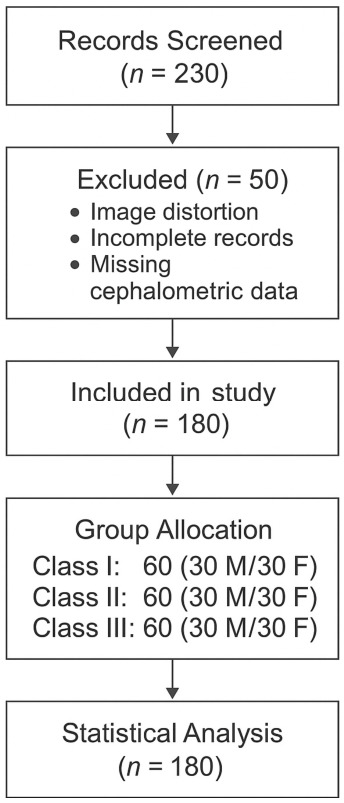
Participant flow diagram of the study.

A total of 230 archived panoramic radiographs were initially screened for eligibility based on predefined inclusion and exclusion criteria. Of these, 50 radiographs were excluded due to image distortion, incomplete records, or missing cephalometric data. The final study sample included 180 patients, evenly distributed across skeletal classes (Class I: *n* = 60; Class II: *n* = 60; Class III: *n* = 60), with 30 males and 30 females in each group (Figure 1). All included patients met the eligibility criteria and were retained for statistical analysis.

Sampling strategy and sample size considerations: From the eligible archive, a stratified sampling approach was used to construct balanced subgroups for the planned factorial comparisons (skeletal class I–III × sex). Specifically, equal numbers were selected for each class (*n* = 60) and sex stratum (30 females and 30 males per class) to support one-way and two-way ANOVA comparisons and to minimize imbalance-related bias. Given the retrospective design, the final sample size was constrained by the number of radiographs meeting strict eligibility criteria within the specified period; adequacy of the achieved sample was subsequently supported by the post hoc power analysis reported below.

#### 2.1.1. Inclusion Criteria

• Age between 16 and 30 years.
• No missing permanent teeth (except third molars).
• Fully erupted maxillary first molars.
• High-quality panoramic radiographs without distortion.

#### 2.1.2. Exclusion Criteria

• Craniofacial syndromes or developmental anomalies.
• History of orthognathic surgery or facial trauma.
• Poor image quality (blurred or distorted radiographs).

Patients were categorized into skeletal Classes I, II, or III based on previously recorded lateral cephalometric tracings. The following cephalometric criteria were used:

• Class I: ANB angle between 0° and 4°.
• Class II: ANB angle > 4°.
• Class III: ANB angle < 0°.

Each skeletal group included an equal number of male and female subjects to assess sex-based differences.

Measurements were made in the infrazygomatic crest (IZC) region, located superior and distal to the apex of the maxillary first molars, approximately halfway between the alveolar crest and the base of the zygomatic arch. All measurements were performed using ImageJ (version 1.54f), a free and validated open-source image analysis software. Prior to analysis, each panoramic radiograph was calibrated using the embedded radiographic scale bar.

### 2.2. Measurement Technique

All measurements were performed using ImageJ software (version 1.54f). Each panoramic radiograph was calibrated to millimeters using the embedded radiographic scale bar. This calibration standardized pixel-to-millimeter conversion for image-based measurements; however, it did not eliminate projection-related magnification, distortion, or head-positioning effects inherent to panoramic radiography.

An occlusal reference line was established by connecting the mesiobuccal cusp tips of the maxillary first molars. When cusp definition was reduced, the closest equivalent occlusal landmarks were used.

Panoramic infrazygomatic crest (IZC) length was defined as the perpendicular two-dimensional linear distance from the occlusal reference line to the most concave point of the zygomatic buttress contour on the panoramic image. This variable was named panoramic IZC length to avoid implying that the measurement represents true three-dimensional bone depth, cortical thickness, sinus proximity, or the oblique extra-alveolar insertion path used clinically for IZC miniscrews.

Measurements were performed separately on the right and left sides, and the bilateral mean value was used as the primary outcome variable. The bilateral mean was selected for the primary subgroup comparison because the objective of the study was radiographic morphometric comparison by skeletal class and sex rather than side-specific miniscrew site planning. Right and left values were first obtained separately before averaging. Therefore, the bilateral mean should not be interpreted as a substitute for side-specific clinical assessment.

**Figure 2:**
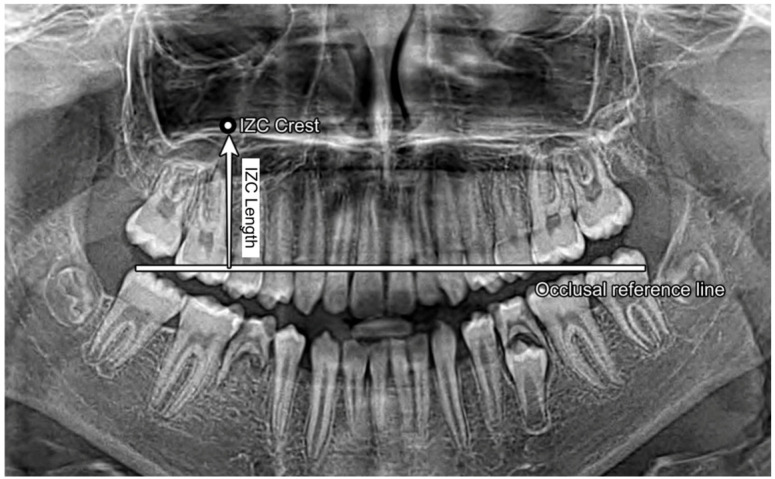
Measurement of panoramic infrazygomatic crest (IZC) length on panoramic radiographs. The occlusal reference line connects the mesiobuccal cusp tips of the maxillary first molars. Panoramic IZC length was defined as the perpendicular two-dimensional radiographic distance from this reference line to the most concave point of the zygomatic buttress contour on the panoramic image. This measurement was used for radiographic morphometric comparison and does not represent three-dimensional bone depth, cortical thickness, sinus proximity, or the oblique extra-alveolar insertion path used for IZC miniscrews.

To improve methodological transparency and reproducibility, the landmark definitions and measurement vector used to quantify panoramic IZC length on panoramic radiographs are illustrated in Figure 2.

### 2.3. Outcomes

The primary outcome variable of this study was panoramic infrazygomatic crest (IZC) length, measured bilaterally from standardized radiographic landmarks on calibrated panoramic radiographs. This outcome represented a two-dimensional panoramic morphometric variable and was not intended to represent three-dimensional bone volume, cortical thickness, sinus relationship, root proximity, or miniscrew insertion trajectory. No secondary outcomes were evaluated in this study.

Although a formal a priori power analysis was not conducted due to the retrospective nature of the study, a post hoc power analysis was performed using G*Power (version 3.1.9.7). Based on the observed effect size for the differences in panoramic IZC length across skeletal classes (effect size f = 0.33), the analysis indicated that a total sample of 180 patients (60 per skeletal class) achieves a statistical power of 0.90 with an alpha level of 0.05 using a one-way ANOVA design. This confirms that the sample size was adequate to detect significant differences with high statistical confidence.

### 2.4. Sources of Bias and Group Comparability

To minimize selection bias, strict inclusion and exclusion criteria were applied, and all radiographs were anonymized prior to measurement. Equal numbers of male and female participants were included in each skeletal class to ensure group comparability. All measurements were performed by a single trained examiner using standardized anatomical landmarks and software calibration procedures. Intra-observer reliability was confirmed by repeated measurements in 10% of the sample, yielding an intraclass correlation coefficient (ICC) of 0.95. Due to the retrospective nature of the study and reliance on archived images, potential sources of bias such as image quality variation or undocumented confounding variables could not be fully eliminated.

Because measurements were performed by a single trained examiner, interobserver reliability could not be assessed. This was considered a methodological limitation and is acknowledged in the Limitations section.

Data were analyzed using SPSS (version 26.0; IBM Corp, Armonk, NY, USA). Descriptive statistics (mean ± SD, minimum, maximum) were calculated for panoramic IZC length. Normality was assessed using the Kolmogorov–Smirnov test, and homogeneity of variances was evaluated with Levene’s test. One-way ANOVA was used to compare panoramic IZC length among skeletal classes, followed by Tukey’s HSD for post hoc pairwise comparisons. Sex differences within skeletal classes were evaluated using independent-samples *t*-tests. A two-way ANOVA (factors: skeletal class [I–III] and sex [female/male]) was performed to test main effects and the sex-by-class interaction. Intra-observer reliability was assessed by repeating measurements in 10% of the sample after two weeks and calculating the intraclass correlation coefficient (ICC). Statistical significance was set at *p* < 0.05.

Effect size estimates were reported to support interpretation of the magnitude of subgroup differences. For the skeletal-class comparison, Cohen’s f and partial eta squared were reported. For sex-based comparisons, Cohen’s d was used where appropriate.

This study adhered to the STROBE (Strengthening the Reporting of Observational Studies in Epidemiology) guidelines for cross-sectional studies to ensure methodological transparency and reporting accuracy.

## 3. Results

### 3.1. Baseline Data

A total of 180 patients were included in the study, with 60 individuals in each skeletal class and equal distribution by sex (30 males and 30 females per class).

The mean age of the participants was 18.2 years (range: 16–30).

### 3.2. Numbers Analyzed and Statistical Estimates

All 180 participants were included in the final analysis. Table 1 presents the mean panoramic IZC length values, standard deviations, minimum and maximum measurements for each skeletal class and sex subgroup. Statistical comparisons were performed using one-way ANOVA, independent samples *t*-tests, and two-way ANOVA to assess interaction effects. Significant differences were found across skeletal classes (*p* < 0.001), between sexes (*p* < 0.01), and for the interaction between sex and skeletal classification (*p* = 0.04). Intra-observer reliability analysis yielded an ICC value of 0.95, indicating excellent measurement consistency.

The mean panoramic infrazygomatic crest (IZC) length values across skeletal classes and sexes are presented in Table 1. Males exhibited greater panoramic IZC length values than females in all skeletal categories. Among the three skeletal groups, Class III individuals demonstrated the highest mean values (5.81 ± 0.38 mm in females and 6.16 ± 0.41 mm in males), while Class II individuals had the lowest values (4.99 ± 0.36 mm in females and 5.51 ± 0.40 mm in males).

**Figure 3:**
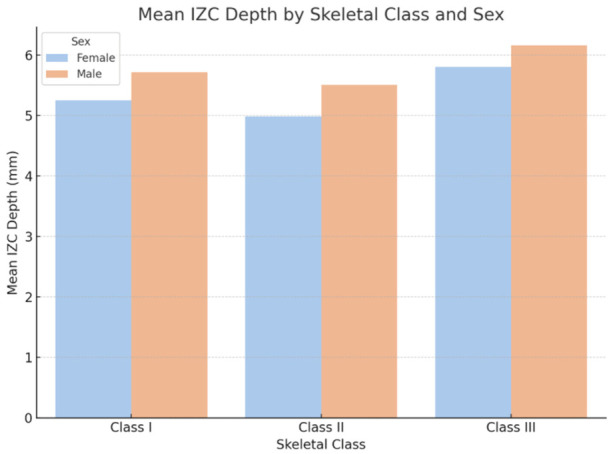
Mean panoramic IZC length by skeletal class and sex. The graph shows average panoramic IZC length values for female and male patients across Class I, Class II, and Class III skeletal categories. Error bars indicate standard deviations. Male subjects showed higher mean values than females in all skeletal groups, and Class III patients showed the highest values overall.

Figure 3 illustrates the mean panoramic IZC length values grouped by skeletal classification and sex. A clear upward trend in panoramic IZC length was observed from Class II to Class III, with consistently higher values in males.

Statistical analysis revealed a significant main effect of skeletal class on panoramic IZC length (one-way ANOVA, *p* < 0.001). Post hoc comparisons (Tukey’s HSD) indicated that Class III had significantly greater panoramic IZC length than both Class I and Class II (Class III vs. Class II: *p* < 0.001; Class III vs. Class I: *p* = 0.02). The difference between Class I and Class II was also statistically significant (*p* = 0.03).

Effect size estimates were added to support interpretation of the magnitude of subgroup differences. The skeletal-class comparison showed a moderate effect size (Cohen’s f = 0.33). The overall male–female difference showed a large effect size based on the summarized group values (Cohen’s d ≈ 0.92). The sex-by-class interaction indicated a small-to-moderate effect size. These estimates suggest that the statistically significant subgroup differences were measurable, although their clinical interpretation should remain cautious because the outcome was a two-dimensional panoramic length variable.

An independent samples *t*-test showed that males had significantly greater panoramic IZC length than females across all skeletal groups (*p* < 0.01). A two-way ANOVA confirmed significant interaction between sex and skeletal class (*p* = 0.04), indicating that sex-based differences in panoramic IZC length varied depending on skeletal pattern.

Measurement reliability was evaluated through intra-observer analysis on 20 randomly selected radiographs. The intraclass correlation coefficient (ICC) for panoramic IZC length measurements was 0.95, indicating excellent repeatability.

## 4. Discussion

This study evaluated panoramic infrazygomatic crest (IZC) length on calibrated digital panoramic radiographs and identified significant differences according to skeletal class and sex. Panoramic IZC length was greatest in skeletal Class III, intermediate in Class I, and lowest in Class II; males also demonstrated greater values than females. These findings indicate that the panoramic radiographic appearance of the IZC region varies across clinically relevant subgroups. However, the measured variable should be interpreted strictly as a two-dimensional panoramic morphometric parameter rather than as true three-dimensional bone depth or an insertion corridor for IZC miniscrews.

The present panoramic measurements should be distinguished from CBCT-derived measurements of bone depth, cortical thickness, sinus proximity, and simulated insertion corridors. CBCT and computed tomographic studies evaluate three-dimensional anatomical structures and may simulate different insertion heights and angulations, whereas the present study measured a perpendicular two-dimensional length on panoramic radiographs. Therefore, the absolute values reported here should not be directly compared with three-dimensional insertion-path measurements. The relevance of the present findings lies mainly in subgroup-based radiographic morphometry and in the preliminary identification of skeletal-class and sex-related differences on panoramic images [10,11,18].

The skeletal class pattern observed in the present study (Class III > Class I > Class II) is consistent with the broader CBCT literature indicating that craniofacial morphology is associated with differences in IZC bone availability and the location of more favorable insertion windows [4,6,7]. Systematic syntheses also underline that heterogeneity of available studies and high variability of bone measurements limit the feasibility of a single universally recommended IZC target site, thereby supporting individualized three-dimensional assessment in selected cases [13]. Taken together, the direction of our findings aligns with anatomic mapping work suggesting that skeletal-pattern differences can translate into measurable variation at the IZC region, although panoramic IZC length alone cannot characterize three-dimensional morphology.

With respect to sex, our finding of greater panoramic IZC length in males is compatible with reports indicating sex-related differences in IZC bone availability in certain populations [19]. However, the current study quantified only panoramic IZC length and did not evaluate cortical thickness, sinus relationship, root proximity, or the three-dimensional insertion path; therefore, clinical risk stratification cannot be inferred from our results [20]. Accordingly, subgroup tendencies observed here should be used for preliminary comparative interpretation rather than deterministic clinical decision-making.

The observed differences between skeletal classes were approximately 0.5–1.0 mm across several subgroup comparisons. These differences may have descriptive value for preliminary radiographic comparison, especially when panoramic records are already available; however, they should not be interpreted as sufficient for miniscrew placement planning. IZC miniscrews are inserted through an oblique extra-alveolar trajectory in close relation to the maxillary sinus and away from the dental root corridor [1,2,3]. For this reason, a single perpendicular two-dimensional measurement on a panoramic image cannot describe the complete bone envelope encountered during placement. In clinical practice, the present measurements may help raise awareness of subgroup variation, but CBCT remains necessary when accurate assessment of insertion trajectory, sinus proximity, cortical thickness, and side-specific anatomy is required [11,13,20,21,22].

The imaging modality is another essential interpretive frame. Panoramic radiography is affected by magnification, distortion, and head-positioning sensitivity, which can influence linear measurements [15,16,17]. Nevertheless, the excellent intra-observer repeatability in this work indicates that, under standardized calibration and landmark definition, panoramic archives can yield reproducible two-dimensional length measurements for retrospective subgroup comparisons. For comprehensive anatomic planning—particularly evaluation of adjacent structures and trajectory—systematic reviews continue to support CBCT use in situations where accurate three-dimensional appraisal is clinically warranted [13]. In addition, meta-analytic work on insertion parameters indicates that bone thickness can vary with vertical insertion height, angulation, and anatomical position, supporting the concept that “where and how” the screw is planned materially affects the available bone envelope [12].

A strength of this study is that it evaluates a standardized panoramic measurement in a balanced sample with equal distribution by skeletal class and sex. Panoramic radiographs are commonly available in orthodontic archives and do not require additional radiation exposure when they have already been acquired for routine diagnostic purposes. Therefore, panoramic-based morphometric assessment may have value for retrospective research and preliminary subgroup comparison. This strength should be understood within the limits of the modality: panoramic imaging can support descriptive radiographic assessment, but it cannot replace CBCT for individualized IZC miniscrew planning.

Future research should directly compare panoramic and CBCT-based IZC measurements within the same individuals, incorporate additional craniofacial variables (including vertical facial type), assess interobserver reliability, and link anatomic metrics to clinical outcomes such as stability and complications. Such studies would clarify how subgroup differences in panoramic-derived IZC length relate to three-dimensional morphology and to clinical performance indicators.

This study demonstrates that panoramic IZC length varies significantly by skeletal class and sex. The results support the use of standardized panoramic measurements for retrospective morphometric comparison and preliminary radiographic assessment. At the same time, the findings should not be extended to claims about true three-dimensional bone availability, sinus safety, root proximity, or miniscrew insertion trajectory. The study therefore contributes descriptive panoramic reference information while reinforcing the need for CBCT when detailed clinical planning is required.

## 5. Limitations

This study has several limitations. First, panoramic radiographs are affected by magnification, projection-related distortion, superimposition, and head-positioning sensitivity, particularly in the posterior maxillary region. Calibration with an embedded scale bar standardized pixel-to-millimeter conversion but did not eliminate these inherent limitations of panoramic imaging. Second, the outcome variable represented panoramic IZC length, a two-dimensional linear measurement, and did not represent true three-dimensional bone depth, cortical thickness, sinus proximity, root proximity, or the oblique extra-alveolar insertion corridor used clinically for IZC miniscrews. Third, right and left measurements were averaged for subgroup-level comparison, which may reduce side-specific clinical interpretability because IZC anatomy can be asymmetric. Fourth, interobserver reliability was not assessed because measurements were performed by a single trained examiner; only intra-observer repeatability was evaluated. Finally, the retrospective single-center design limited control over potential confounders and did not allow assessment of clinical outcomes such as miniscrew stability, complications, or sinus-related findings.

## 6. Conclusions

Panoramic IZC length measured on calibrated panoramic radiographs differed significantly according to skeletal class and sex, with higher values in skeletal Class III individuals and males. These findings provide preliminary comparative morphometric reference information from routinely available panoramic records. However, panoramic IZC length is a two-dimensional radiographic measurement and should not be interpreted as a true three-dimensional bone depth measurement or as a direct guide for IZC miniscrew insertion. CBCT remains necessary when comprehensive assessment of sinus proximity, cortical thickness, insertion angulation, extra-alveolar trajectory, and side-specific anatomy is clinically required.

## Figures and Tables

**Table 1:** Panoramic IZC Length Measurements. Mean, standard deviation, minimum, and maximum panoramic IZC length values by skeletal class and sex.

Skeletal Class	Female Mean ± SD (mm)	Female Min–Max (mm)	Male Mean ± SD (mm)	Male Min–Max (mm)	*n* (F/M)
Class I	5.25 ± 0.37	4.52–6.04	5.72 ± 0.36	5.03–6.43	30/30
Class II	4.99 ± 0.36	4.23–5.99	5.51 ± 0.40	4.45–6.13	30/30
Class III	5.81 ± 0.38	5.22–6.79	6.16 ± 0.41	5.56–7.08	30/30

## Data Availability

The original contributions presented in this study are included in the article. Further inquiries can be directed to the corresponding author.
